# Enveloped RNA virus utilization of phosphatidylserine receptors: Advantages of exploiting a conserved, widely available mechanism of entry

**DOI:** 10.1371/journal.ppat.1009899

**Published:** 2021-09-23

**Authors:** Dana Bohan, Wendy Maury

**Affiliations:** 1 Department of Microbiology and Immunology, University of Iowa, Iowa City, Iowa, United States of America; 2 Interdisciplinary Immunology Graduate Program, University of Iowa, Iowa City, Iowa, United States of America; Mount Sinai School of Medicine, UNITED STATES

Enveloped RNA virus entry is a conceptually simple stepwise process: A virus attaches to host cells, which leads to viral membrane/cellular membrane fusion and viral genome injection into the cytoplasm. The specifics of and intermediate steps during this entry process are complex and vary widely among viruses. Some of these viruses attach and fuse directly with the plasma membrane, whereas others enter endosomes following attachment and subsequently fuse with endosome membranes. The nature of the host/enveloped RNA virus interactions that lead to virion/cell membrane fusion can also vary. With some viruses, the viral glycoprotein specifically and directly binds with high affinity to a cell surface receptor that mediates the fusion process. At the other end of this spectrum are viruses that use broader, less selective mechanisms for cellular attachment. Two commonly used broad mechanisms employed by enveloped RNA viruses are the binding of virion glycoprotein-associated glycans to glycan-binding proteins such as C-type lectins and the binding of virion lipids such as phosphatidylserine (PS) to PS receptors. These glycoprotein-agnostic attachment factors not only attach virus to the surface of cells, but frequently mediate virion internalization to endosomes. Importantly, they do not mediate membrane fusion. Thus, for viruses that use their glycans and/or lipids as attachment/internalization factors, additional entry steps, such as binding within the endosomal compartment to a cellular receptor that stimulates fusion events, are required for productive infection.

The ability of glycans on virion glycoproteins to enhance attachment has been long appreciated [1–4]; however, the importance of virion-associated PS binding of host cell PS receptors has more recently demonstrated [5–8]. Initial studies demonstrated that PS on the surface of the DNA virus, vaccinia virus (VACV), mediated virus binding and internalization into endosomes via an actin-dependent macropinocytosis-like event [8]. The authors insightfully identified this virus uptake mechanism as similar to apoptosis and termed it “apoptotic mimicry.” However, the receptors mediating this uptake were not identified in this study. Subsequently, RNA viruses in the Filoviridae and Flaviviridae families were identified to use PS receptors of the TIM and TAM families [5,6,9] and that virus interaction with these receptors was through virion-associated PS [7,10–12]. The number of RNA virus families that utilize this uptake mechanism has increased steadily, with additional members of the Bunyaviridae and Arteriviridae most recently identified [13,14]. Yet, PS has relatively low affinity for PS receptors compared to the affinity of many viral glycoproteins for their cognate receptors. This begs the question: What advantages are conferred to viruses by utilization of PS receptors? This review discusses the fundamentals of virion PS/PS receptor interactions and highlights the numerous benefits conferred by this route of entry.

## Enveloped virus utilization of PS receptors: Tools of the trade

PS is a ubiquitous negatively charged phospholipid of cellular membranes that is critical for cellular debris recycling and cargo trafficking. In healthy cells, PS is retained on the cytoplasmic (or inner) leaflet of the plasma membrane at high energetic cost by flippases [15]. Scramblases, in response to cell death signals or transient Ca^2+^ fluxes, translocate PS to the outer plasma membrane leaflet of apoptotic bodies where exposed PS binds to and is internalized by PS receptors on phagocytic or endocytic cells. This leads to recycling of cellular constituents [16]. PS receptors are widely and highly expressed in a variety of tissues, being critical factors in ensuring efficient, rapid, and minimally inflammatory clearance of apoptotic cells [17].

The function of PS receptors is readily co-opted by enveloped viruses as many present PS on the surface of their lipid envelope (for earlier reviews, see [18–20]). The ubiquitous nature of PS in cellular membranes combined with a panoply of PS receptors encoded by mammalian hosts combine to make this a near-universally available mechanism for enveloped virus/host cell interaction. A wide range of pathogenic viruses are appreciated to exploit PS receptors in mammals (**Table 1**), including filoviruses (Ebola virus (EBOV) and Marburg virus), flaviviruses (Dengue virus (DENV), West Nile virus (WNV), Yellow Fever virus, and Zika virus (ZIKV)), poxviruses (VACV), alphaviruses (Chikungunya virus (CHIKV), Sindbis virus, and Eastern Equine Encephalitis virus), bunyaviruses, (Hantaan virus and Andes virus), and arenaviruses (Pichinde virus (PICV)) [6,8,10,13,21–24]. Recent research from our lab suggests that this extends to coronaviruses as well, as Severe Acute Respiratory Syndrome Coronavirus 2 (SARS-CoV-2) utilizes PS receptors to enhance entry [25]. Even nonenveloped viruses such as the picornaviruses hepatitis A virus (HAV) and enteroviruses as well as orthohepevirus hepatitis E virus (HEV) utilize apoptotic mimicry (infrequently termed “exosome mimicry” in these contexts) [26–28]. These nonenveloped viruses are incorporated into and circulate in vivo within lipid membranes rich in PS. These quasi-enveloped virions are taken up by cells, allowing PS-dependent virus spread in vivo.

**Table 1:** PS receptors that enhance enveloped virus infection: expression and functionality.

PS Receptor (*GENE*)	Cell Types Expressing mRNA*	Viral Families Known to Utilize for Entry^#^	Notes
TIM-1 (*HAVCR1)*	Some T cells and B cells, renal epithelia, colon epithelia; syncytiotrophoblasts; numerous commonly used epithelial lines	filoviruses; alphaviruses; flaviviruses; arenaviruses, poxviruses; baculoviruses	Broad uptake of apoptotic mimicking viruses High expression in damaged renal epithelia
TIM-4 (*TIMD4*)	Tissue macrophages such as Kupffer cells, peritoneal macrophages, adipocyte macrophages; testicular germ cells	filoviruses; flaviviruses; alphaviruses; arenaviruses	Broad uptake of apoptotic mimic viruses Constitutive, high expression on a variety of different tissue macrophages; extremely high expression in testes
AXL (*AXL*)	B cells; endothelia; tissue macrophages such as alveolar macrophages and Kupffer cells; fibroblasts, epithelia, smooth muscle cells, Sertoli cells	filoviruses; alphaviruses; flaviviruses; arenaviruses, poxviruses; baculoviruses; coronoviruses	Broad uptake of apoptotic mimic viruses; high and broad cellular expression; ubiquitous ligands (Protein S and Gas6); multifunctional kinase domains
MerTK (*MERTK*)	Tissue macrophages such as Kupffer cells and peritoneal macrophages; Ito cells; lung epithelia; syncytiotrophoblasts; retinal rod photoreceptor cells		
Tyro3 (*TYRO3*)	Renal peritubular cells; Leydig cells; syncytiotrophoblasts; keratinocytes; fibroblasts		
CD300A (*CD300A*)	Macrophages such as Kupffer cells; granulocytes; T cells; B cells; lung epithelia	flaviviruses	Selective uptake of apoptotic mimicking viruses Broad immune cell expression Binds to PS and PE

*Cell type mRNA expression determined by The Human Protein Atlas scRNAseq data (https://www.proteinatlas.org/humanproteome/celltype).

^#^Virus families utilizing denoted PS receptors were identified from Moller-Tank and colleagues [20].

PE, phosphatidylethanolamine; PS, phosphatidylserine.

PS receptors vary widely both in structure and expression patterns, but all bind PS either directly or indirectly [29]. Two families of PS receptors seem to be most important for enveloped RNA virus uptake into cells: the TIM and TAM receptors (**Table 1**) [7,11,12,30]. The TAM tyrosine kinase receptor family, Tyro3, AXL, and MerTK do not bind to PS directly. Instead, they bind to the adapter proteins Gas6 or Protein S that, in turn, bind to PS. Formation of the PS-containing complex initiates a signaling cascade by the TAM kinase domain, which mediates internalization of the complex into endosomes. Two TIM (T-cell immunoglobulin and mucin domain) family members, TIM-1 and TIM-4, bind PS directly through a PS binding pocket in an N-terminal immunoglobulin V (IgV)-like domain [31,32]. While TIM-1 has been shown to internalize its bound cargo into the endosome [11,12], TIM-4 has modest, but significant internalization capabilities [30,33] that are enhanced by interactions with the Fn III domain of integrins or MerTK [33–38]. In addition to the 2 dominant PS families, the lactadherin MFG-E8, which binds PS and is internalized by integrins αvβ3 or αvβ5, and CD300a mediate uptake of some enveloped viruses [23]. Additionally, the membrane lipid phosphatidylethanolamine (PE) functions similarly to PS in the context of viral apoptotic mimicry, enhancing entry for DENV, WNV, and EBOV by interactions with TIM-1 and CD300a [11,24].

Major cell types expressing TIM-1 include some T cell and B cell populations, renal epithelia, and a wide variety of epithelial cell lines, such as Vero cells and Huh7 cells. TIM-4 expression is expressed on a variety of tissue macrophages, including Kupffer cells and large peritoneal macrophages. MerTK is also expressed on macrophages, as well as NK, NKT, and platelets. AXL expression is broad, found on a variety of epithelia, endothelial, fibroblasts, macrophages, and other connective tissue cells, whereas Tyro3 is more strongly localized to brain tissue. These expression patterns likely play a role in the cell tropism of viruses that use PS receptors for binding and internalization. Interestingly, viruses seem to preferentially use one PS receptor over others. TIM-1 is preferentially used by EBOV, CHIKV, and DENV, whereas SARS-CoV-2 or ZIKV entry is preferentially enhanced by AXL [7,25,39,40]. The mechanism driving this distinction is unknown.

## Enveloped virus utilization of PS receptors: Mechanisms of PS incorporation

Mechanisms of PS acquisition are as diverse as the apoptotic mimicking viruses themselves. Some viruses such as EBOV have strategies that enhance incorporation of PS into their viral envelope. The scramblase XKR8 that is activated during apoptosis colocalizes with EBOV matrix (VP40) and glycoprotein (GP) within producer cells and XKR8 knockout cells produce PS-deficient virions [41,42]. Scramblase colocalization with EBOV glycoprotein suggests that PS incorporation into filoviruses is a virus-facilitated process. Another established mechanism by which viruses, e.g., PICV, may acquire PS is through rapid triggering of apoptosis and subsequent scramblase activation and flippase deactivation, followed by budding from the freshly PS-decorated plasma membrane [43]. The presence of PS on the outer leaflet of viral envelopes may not always be an active process. Enveloped viruses bud from PS-rich membranes such as the endoplasmic reticulum or plasma membrane and equilibration of PS on the inner and outer leaflets of viral envelopes may occur over time as maintenance of the nonequilibrium state requires both cellular flippases and ATP.

## Enveloped virus utilization of PS receptors: Advantages and disadvantages of this functionally conserved mechanism

One common viral lifestyle strategy that is benefited by the use of PS receptors is virus targeting of a wide variety of host species. Flaviviruses that must enter and replicate in arthropod and mammalian cells are excellent examples. With host evolution and speciation, sequences of specific cellular receptors diverge due to positive and stochastic selection events, thereby narrowing rather than expanding the host range for viruses due to host receptor divergence. Apoptotic mimicry is a strategy that avoids these evolutionary constraints, as PS does not evolve and a range of PS receptors are available, which, as noted below, frequently are highly conserved across host species.

**Fig 1:**
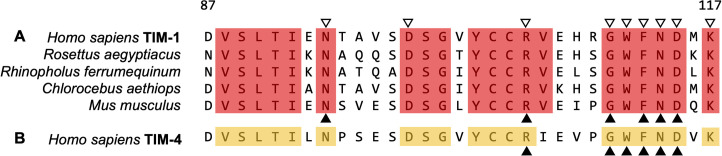
The TIM-1 PS binding pocket is highly conserved across a wide range of mammalian genomes. **(A)** TIM-1 amino acid sequences from NCBI were accessed for human, Egyptian fruit bat, greater horseshoe bat, African green monkey, and house mouse and aligned using CLUSTAL Ω. A region of 30 residues is shown here, encompassing the IgV binding pocket. Highlighted residues have complete identity across all species examined, ▽ depict residues that, if mutated (to alanine), decrease EBOV pseudovirion transduction >20%, and ▲ indicates residues that, if mutated, decrease EBOV GP-rVSV infection >20% according to Moller-Tank and colleagues [45]. **(B)** Alignment of TIM-4 amino acid sequence to TIM-1 as above. Highlighted residues have identity to all TIM-1 sequences shown, and ▲ indicates residues that, if mutated, significantly reduce transduction and infection according to Rhein and colleagues [30]. EBOV GP-rVSV, Ebola virus glycoprotein-recombinant vesicular stomatitis virus; IgV, immunoglobulin V; PS, phosphatidylserine.

As an extension of this, apoptotic mimicry has clear implications for zoonotic cross-species transmission, namely the potential for spillover events. The highly conserved nature and broad expression patterns of different PS receptors provide an enticing route of entry/internalization compatible for crossing between distantly related species. For successful spillover events to be potentiated via PS receptors, viral glycoproteins must mediate membrane fusion events in these different species, which are, by no means, guaranteed and thus are a primary determinant in host range. However, alignments of PS receptor orthologs across mammalian species frequently demonstrate strong sequence homology/identity. For the TIM receptors, the N-terminal IgV-like domain directly binds to virion-associated PS through interactions with amino acids located within a PS binding pocket [44,45], although additional amino acids outside the pocket also contribute to PS binding [30,46,47]. The TIM IgV PS binding pocket (RGWFNDMK) is highly conserved, with striking similarity/identity of these sequences between the human encoded residues and those of the horseshoe bat and fruit bats (**Fig 1A**). These bats are closely related to suspected SARS-CoV and EBOV reservoirs, respectively. The PS binding pocket of TIM-1 is not only shared across mammalian species, but most members of the TIM family also share a striking conservation of this pocket (**Fig 1B**), with nearly identical functional residues present in TIM-1, TIM-3, and TIM-4, a series of clear systemic redundancies [30]. Given the use of this conserved virus internalization mechanism, it is somewhat surprising that deletion of one or more of the PS receptor families to reduce virus infection of mammalian hosts is not more frequently observed. This suggests that the indispensable nature of PS receptor activity far exceeds the detrimental effects of enhanced viral infection. A notable exception is the deletion of TIM-1 in portions of the New World monkey family tree, indicating that this PS receptor is dispensable in specific contexts [48]. Further study of evolutionary origins, links, and conservation of this important family of receptors is needed.

Not only does utilization of PS receptors allow viruses to target multiple species, but the conserved nature of PS allows multiple PS receptor families to interact with the same ligand to expand the range of permissive cell types within a single host. Even with viruses that interact with high-affinity cognate receptors, PS receptors expand viral tropism by mediating entry into cells that lack expression of that receptor on the surface. For instance, while Lassa virus (LASV) is well characterized to bind and internalize into endosomes through high-affinity interactions with the host protein α-dystroglycan (DAG), loss or aberrant glycosylation of DAG results in LASV being trafficked into the endosomal compartment by PS receptors such as TIM-1, rather than DAG, where interaction with endosomal LAMP1 mediate fusion [49]. The ability of PS receptors to expand the tropism of other viruses with similar entry strategies may also occur, but is poorly studied. It is crucial to note that viral tropism is multifactorial, with the additional requirement of viral glycoprotein interactions with a cognate cellular receptor in most cases.

PS-dependent virus uptake is also advantageous for viruses by allowing protection of viral glycoprotein receptor binding sites from antibodies in the extracellular environment. It is now well documented that neutralizing antibodies are not produced against the receptor binding motif of EBOV glycoprotein [50]. The absence of these targeted neutralizing antibodies is thought to be due to the sequestration of this motif in the extracellular glycoprotein, making it unavailable as a target for antibodies. The EBOV receptor binding motif is only surface exposed and available for cognate receptor binding (or antibody neutralization) once the virion is internalized into endosomes and proteolytically processed. Thus, PS-dependent uptake of EBOV virions allows the receptor binding motif to be sheltered extracellularly. Whether this applies to any other virus is unexplored.

One notable feature of PS receptor internalization and recycling of apoptotic bodies is a dampening of inflammation, as failure to clear apoptotic cells can elicit robust innate immune responses including proinflammatory cytokines and chemokines [51]. PS receptor uptake of apoptotic bodies results in signaling events that reinforce an anti-inflammatory state by both promoting anti-inflammatory cytokine secretion and suppressing inflammatory cytokine transcription [52,53]. This “quiet entry” was first noted by Mercer and Helenius, observing that PS-laden VACV entry was minimally inflammatory [8]; however, the role of PS receptors in this anti-inflammatory state was unappreciated at the time. Later, genetic ablation of the 3 TAMs in murine cells was found to dramatically increase production of antiviral type I interferons following flavivirus and lentivirus entry, suggesting that the immunosuppressive state driven by these receptors is advantageous to viral pathogens [54]. Thus, the anti-inflammatory nature of PS receptor internalization serves to further benefit apoptotic mimics by decreasing the likelihood of virus restriction, inhibition, and destruction by host innate immune responses [17].

While the inherent low-affinity interactions between PS and PS receptors is likely a disadvantage for a broad range of viruses to use this uptake mechanism, specific viruses are directly antagonized by PS receptors. For instance, TIM-1 serves as a restriction factor for human immunodeficiency virus (HIV) [55]. Following the budding of lentiviral particles, TIM-1 binds and prevents complete release of virions via incorporation into the HIV envelope [56]. This retention phenotype reduces particle infectivity 100-fold, is enhanced by SERINC proteins, and is antagonized by viral accessory protein Nef. It is curious that TIMs are decidedly antiviral for HIV, but not for other enveloped viruses [56]. One possible explanation for this is that HIV enters cells through high-affinity interactions with specific surface receptors. Hence, during HIV infection, PS receptors are not internalized from the plasma membrane during infection. Thus, upon virus egress, those receptors remain abundant on the plasma membrane and therefore available to bind and restrict virion release.

## Enveloped virus utilization of PS receptors: Challenges, opportunities, and therapeutic promise

The therapeutic value of virus/PS receptor blockade has been studied in the context of several viral pathogens. Administration of PS binding antibodies to PICV-infected guinea pigs reduced mortality after infection and facilitated macrophage killing of infected fibroblasts [57]. Another antiviral approach targeting PS receptors is the use of a soluble TIM-1 IgV domain construct [58]. This construct reduces ZIKV infection of human and, notably, mosquito cells through competitive binding of virion PS. The authors show the construct’s ability to block EBOV pseudovirion infection as well, demonstrating the virus-agnostic, host-agnostic, PS-dependent mechanism [58]. Small molecule inhibitors against PS receptors signaling and internalization are also available. We have recently shown that in AXL-expressing lung cell lines an AXL-specific inhibitor reduces SARS-CoV-2 loads and infectious titers [25]. Further, anti-PS receptor antibodies are available that block virus/PS receptor interactions [6,12].

The foremost obstacles to therapeutically impeding apoptotic mimicry are PS receptor redundancy and alternative viral strategies of entry. However, the in vivo studies performed to date suggest that inhibition of specific PS receptors may be efficacious [59]. Concerns remain regarding suppression of the physiological role of PS receptors, yet this topic is understudied. Encouragingly, AXL inhibitors are currently in multiple clinical trials for certain metastatic cancers, suggesting a reasonable safety profile. This is an area of antiviral research that will likely prove fruitful in the future. As PS, PS receptor structure, and PS receptor function are highly conserved attributes among mammalian hosts and functionally conserved in insect hosts, the potential of PS receptors to facilitate zoonotic transmission under specific circumstances should not be ignored. PS receptors are an armory of double-edged swords for the host, clearing debris in an immunological quiescent manner, but also offering an open door to appropriately prepared viral pathogens.
